# Travis County Medical Society

**Published:** 1914-12

**Authors:** 


Travis County Medical Society.—What was declared to be one of the best attended and most profitable meetings of the Travis County Medical Society this year was that held in the Driskill Hotel Friday, October 13th. This was the monthly meeting, and twenty-seven members attended.

The proposition of making plans for the meeting of the District Medical Society to be held in Austin about December 22d was discussed. About one hundred physicians are expected to attend. The district includes the counties of Travis, Williamson, Bastrop, Burnet, Llano, Mason, Blanco, Hays, Colorado and Lee. There are about three hundred physicians in this district who are members of the society.

One of the most interesting cases ever reported to the Travis County Medical Society was discussed by Dr. Key, who told of a patient who had lost his vision because of excessive use of alcohol and tobacco. It was stated that the man had for some time used one or the other of the narcotics every hour except when he was busy eating or sleeping. The case was discussed by Dr. Harper.

Dr. P. E. Suehs reported a case of secondary hemorrhage from tonsilotomy. This case was discussed by Drs. Harper, Smart, Key, Eckhardt and Hill. Dr. Joe Gilbert reported a case of congenital pyloric stenosis, which was discussed by Drs. McLaughlin and Bennett. Dr. Gilliland reported a case of actinimycosis, which was discussed by Drs. Bennett, Scott, Smart and McAdams.

Dr. Murray, Assistant City Physician, told the doctors about the new City Hospital, and invited them to hold a meeting at the hospital when it it completed some time in December.

Those present at the meeting were: Drs. Mathis, Granberry, Hill, Smart, Decherd; Vaughan of Liberty Hill; Bennett, Graves, Maxwell, Bibb, Davis, McAdams, Key, Beverly, McLaughlin, Sterzing, Krueger, Harper, Hudson, Gilbert, Nichols, Eckhardt, Suehs, Murray, Scott and Gilliland.

---

Delta County Medical Society held its November meeting in the directors' room of the First National Bank, of Cooper, on November 2d, with a good attendance. Dr. O. Y. Janes read his paper, "The Business Side of Medicine," and the subject was discussed at length.

---

The Fourth District Medical Society was held in Ballinger November 3d and 4th. After speeches of welcome had been delivered by Marcellus Kleberg and Dr. T. E. Mangum, of Ballinger, and a response by Dr. R. H. Cochran, of Coleman, the afternoon session was begun by President Dr. John W. Ellis, of Lampasas, who delivered the annual address.

The afternoon session was made up of sixteen papers read by the visiting physicians on "General Medical Practice."

Wednesday the surgical section was taken up, while the eye, ear, nose and throat section closed the convention.

One of the features of the social program was an immense banquet, which was tendered the visiting doctors and friends at the Central Hotel in the evening. Dr. W. B. Halley, of Ballinger, acted as toastmaster for the occasion. During the afternoon automobiles took the guests for a tour of the city and the territory adjacent to the city itself.

One of the most prominent physicians in the United States, Dr. Paul Gronerrud, of Chicago, was in attendance at the meeting, and read a paper before the body. It was in Dr. Gronerrud's honor that the banquet was spread.

San Angelo was named as the 1915 meeting place. Officers elected were: J. M. Horn, Brownwood, President; R. H. Cochran, Coleman, Secretary-Treasurer.

---

The regular session of the McLennan County Medical Society was held in the rooms of the Chamber of Commerce.

The following scientific program was carried out: "Scope and Limitations of X-ray Therapy," Dr. R. B. Nutter. Discussion by Dr. I. E. Colgin; "Treatment of Hypertension," Dr. W. S. Witte. Discussion by Drs. A. M. Curtis and W. F. Curran. Dr. Nutter is Chairman of the Program Committee.

---

The Falls County Medical Association met in the Baptist Church in Lott November 2d. The meeting was called to order by the President, Dr. Buie, of Marlin, and the regular routine of business was gone through.

Two very interesting papers were read, one on "Anesthesia," by Dr. Geo. S. McKelvey, and the other one on "Fractures," by Dr. Sherwood.

The meeting was well attended and was one of enthusiasm. The next meeting will be held in Marlin, the first Monday in December.

---

The Brown County Medical Society met in regular monthly session Tuesday, October 10th. Besides a good attendance of Brownwood doctors, several visiting physicians met with the society; included in their number were Dr. Dudgeon, of Sweetwater; Dr. Nichols, of Bangs, and Dr. Strozier, of Santa Anna.

Several interesting papers were read, and a number of discussions were much enjoyed. Considerable business was transacted. Altogether, the meeting is said to have been one of the best the doctors have held in some time.

